# Survival prediction of high-risk outborn neonates with congenital diaphragmatic hernia from capillary blood gases

**DOI:** 10.1186/s12887-016-0658-y

**Published:** 2016-07-29

**Authors:** Ruža Grizelj, Katarina Bojanić, Ena Pritišanac, Tomislav Luetić, Jurica Vuković, Toby N. Weingarten, Darrell R. Schroeder, Juraj Sprung

**Affiliations:** 1 Department of Pediatrics, University of Zagreb, School of Medicine, University Hospital Centre Zagreb, Zagreb, Croatia; 2 Department of Obstetrics and Gynecology, University Hospital Merkur, Zagreb, Croatia; 3 Department of Pediatric Surgery, University of Zagreb, School of Medicine, University Hospital Centre Zagreb, Zagreb, Croatia; 4 Department of Anesthesiology, Mayo Clinic, 200 First St SW, Rochester, MN 55905 USA; 5 Division of Biomedical Statistics and Informatics, Mayo Clinic, 200 First St SW, Rochester, MN 55905 USA

**Keywords:** Congenital diaphragmatic hernia, Outcome, Survival, Clinical prediction, Risk assessment, Capillary blood gases, Outborn

## Abstract

**Background:**

The extent of lung hypoplasia in neonates with congenital diaphragmatic hernia (CDH) can be assessed from gas exchange. We examined the role of preductal capillary blood gases in prognosticating outcome in patients with CDH.

**Methods:**

We retrospectively reviewed demographic data, disease characteristics, and preductal capillary blood gases on admission and within 24 h following admission for 44 high-risk outborn neonates. All neonates were intubated after delivery due to acute respiratory distress, and were emergently transferred via ground ambulance to our unit between 1/2000 and 12/2014. The main outcome measure was survival to hospital discharge and explanatory variables of interest were preductal capillary blood gases obtained on admission and during the first 24 h following admission.

**Results:**

Higher ratio of preductal partial pressure of oxygen to fraction of inspired oxygen (PcO_2_/FIO_2_) *on admission* predicted survival (AUC = 0.69, *P* = 0.04). However, some neonates substantially improve PcO_2_/FIO_2_ following initiation of treatment. Among neonates who survived at least 24 h, the highest preductal PcO_2_/FIO_2_ achieved in the initial 24 h was the strongest predictor of survival (AUC = 0.87, *P* = 0.002). Nonsurvivors had a mean admission preductal PcCO_2_ higher than survivors (91 ± 31 vs. 70 ± 25 mmHg, *P* = 0.02), and their PcCO_2_ remained high during the first 24 h of treatment.

**Conclusion:**

The inability to achieve adequate gas exchange within 24 h of initiation of intensive care treatment is an ominous sign in high-risk outborn neonates with CDH.

We suggest that improvement of oxygenation during the first 24 h, along with other relevant clinical signs, should be used when making decisions regarding treatment options in these critically ill neonates.

## Background

The presence of respiratory distress in neonates with congenital diaphragmatic hernia (CDH) may indicate severe pulmonary hypoplasia, which is a major contributor to morbidity and mortality. In neonates with CDH (i.e., without a concomitant major anomaly or genetic defect), an early presentation of respiratory distress is related to an increased degree of pulmonary hypoplasia [1, 2]. Accurate prediction assessment tools for outcomes of these patients are important because they may be used to rationalize resource management [3–6], guide family counseling, and set benchmarks for severity-based stratification in research [7].

Several newborn illness severity and mortality risk scores are available for CDH neonates [7–10]. For predicting risk of death, the Congenital Diaphragmatic Hernia Study Group (CDHSG) used a logistic regression equation based on birth weight and 5-min Apgar scores [8]. The Wilford Hall/Santa Rosa clinical prediction formula (WHSRPF), uses arterial blood gas values during the initial 24 h of life to estimate survival [7]. The inability to achieve certain levels of pulmonary gas exchange is a good marker of the extent of lung hypoplasia and is associated with increased mortality [11–14]. One study found that *admission* preductal hemoglobin saturation predicts survival [15]. However, *admission blood gas* measurements may be an imprecise indicator of true lung function because these might *reflect suboptimal ventilation during patient transfer from a local maternity ward to a referral center (outborns)*. When considering the outborn CDH neonates with high acuity, we hypothesized that several hours of postadmission ventilation may unmask children with efficient vs. non-efficient gas exchange, and therefore that blood gases obtained after treatment is initiated may increase the prognostication accuracy.

We report a group of high-risk CDH neonates who were tracheally intubated and ventilated before being transferred from either local or remote hospitals to our center for treatment. We examined their capillary blood gases immediately on admission and subsequent blood gases during the first 24 h after admission to determine their predictive role in survival.

## Methods

This study was approved by the Institutional Ethics Committee of the University Hospital Centre (UHC), Zagreb, Croatia. In this retrospective study design, written consent was waived by the Institutional Ethics Committee, and a single approval was obtained for the retrospective chart review.

### Study setting

The UHC is the largest Croatian tertiary referral center for neonatal care, but it does not have a maternity ward. Other Croatian hospitals that do have maternity wards do not have the capacity to manage high-risk neonates in need of neonatal surgery. Therefore, all neonates with CDH in this series are outborns.

### Patient population

This cohort represented CDH outborns with early respiratory distress (i.e., immediately after birth) requiring emergent tracheal intubation between January 1, 2000, and December 31, 2014, who survived transfer to the UHC for the initiation of intensive care management. Excluded were low-risk neonates with late presentation of respiratory distress and neonates without respiratory distress who received elective intubation for corrective surgery, those with concomitant lethal congenital anomalies, and moribund neonates who received resuscitation on admission but died before intensive care management was instituted.

### Neonatal transport management

All neonates were outborns and were transported to the UHC via ground ambulance, sometimes from remote country regions. During transport 9 (20 %) neonates received ventilation through a pressure controlled ventilator integrated in transport incubator (8/13 from distant hospitals in the country, and 1/31 from a local hospital). In the other 35 (80 %), ventilation during transfer was accomplished via hand-held self-inflating bags.

### Neonatal intensive care management

Since January 2000 all neonates with CDH treated at UHC received protective ventilation aimed to minimize volutrauma with the use of minimal pressure and volume settings and inspired oxygen concentration set to achieve acceptable preductal oxygenation saturations (≥85 %) while permitting hypercapnia (≤65 mmHg). Two modes of ventilation were used: assist-control plus volume limit setting mode (*n* = 19) and pressure support ventilation with volume guarantee mode (*n* = 25). Details of ventilatory strategies used during the time frame of this study were recently reported [16]. With this protective ventilation strategy, sedation and muscle paralysis were infrequently used and only in newborns with patient-ventilator asynchrony. High-frequency oscillation ventilation was a rescue treatment for neonates who continued to have hypoxia and hypercarbia (PcCO_2_ > 65 mmHg) despite optimization of protective ventilation. Inhaled nitric oxide (iNO) was used for neonates with echocardiographic findings of pulmonary hypertension. Surgical repair via laparotomy was done following the initial optimization of respiratory parameters, systemic blood pressure, and pulmonary hypertension. Neonatal extracorporeal membrane oxygenation (ECMO) was not available in Croatia during the study timeframe.

### Study design and data collection

This study was a retrospective chart review. We reviewed demographic and birth information (sex, gestational age, birth weight, and Apgar scores); comorbid conditions (pulmonary hypertension assessed with Doppler echocardiography and defined as pulmonary artery pressure to systemic arterial pressure ratio >2/3); CDH information (prenatal diagnosis, type of CDH, presence of peritoneal sac or diaphragmatic agenesis); preductal capillary blood gas data obtained at admission and best and worst preductal capillary blood gas results within the first 24 h after admission but before surgical intervention; and, within 12 h of admission, the lowest body temperature and mean systemic blood pressure. Instead of measuring arterial blood gases, the UHC neonatology intensive care unit (NICU) measures blood gases using capillary blood, and oxygenation is monitored by pulse oximetry. It has been shown that capillary blood gases accurately reflect arterial pH and PaCO_2_ in pediatric intensive care unit patients [17]. Capillary blood gases less accurately reflect arterial PO_2_ in normoxia and hyperoxia, but the accuracy improves under hypoxic conditions [18].

Other abstracted variables related to CDH management included the use of iNO, surfactant, and vasoactive support, and the type of surgical repair (primary or non-primary with patch). The probability of survival (POS) was calculated from the equation proposed by the CDHSG [8] and neonates were categorized into three groups: low POS (0-33 %), moderate POS (34-66 %), and high POS (67-100 %).

### Statistical analysis

The main outcome measure was survival to hospital discharge and the explanatory variables of interest were preductal capillary blood gas values from the first 24 h. Data are presented as mean ± standard deviation for continuous variables and frequency (percentage of sample) for categorical variables. Differences between survivors and nonsurvivors were compared with the independent samples *t*-test for continuous variables and with the *χ*2 test or Fisher exact test for categorical variables. Logistic regression and receiver operating characteristic (ROC) curve analyses were also performed and summarized as the area under the curve (AUC) with sensitivity, specificity, positive predictive value (PPV), and negative predictive value (NPV), with cutoffs determined as the observed value that minimizes the function [(1 − Sensitivity) 2 + (1 − Specificity) 2]^½^. In all cases, 2-tailed *P* values <0.05 were considered statistically significant. Data were analyzed with SAS version 9.3 software (SAS Institute Inc, Cary, NC).

## Results

### Study population

**Fig. 1:**
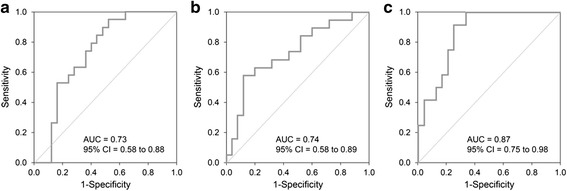
Receiver Operating Characteristic Curve and Area Under the Curve (AUC) for: **a** Probability of survival score (POS) for all neonates. **b** Partial pressure of carbon dioxide in the preductal capillary blood (PcCO_2_) for all neonates on admission. **c** Highest ratio of partial pressure of oxygen in the preductal capillary blood to fraction of inspired oxygen (PcO_2_/FIO_2_) during the first 24 h after admission for neonates who survived more than 24 h after admission

Between January 1, 2000, and December 31, 2014, we treated 66 neonates with CDH. Of these, 13 were low risk CDH (asymptomatic), and 53 high-risk CDH neonates presented with early respiratory distress after delivery. We excluded 9 of the 53 patients: 1 with lethal trisomy 18 (Edward’s syndrome), 1 with missing medical records, 1 with surgery performed in a remote hospital transferred to our unit for further treatment, and 6 moribund neonates who were briefly resuscitated but died during admission. Demographic data, disease characteristics, and therapeutic interventions for the remaining 44 high-risk neonates were summarized for survivors (*n* = 25) and nonsurvivors (*n* = 19) (Table 1). As expected, neonates who died had worse 1-min and 5-min Apgar scores, lower calculated POS scores, and a higher frequency of pulmonary hypertension and diaphragmatic agenesis. To further characterize the value of the POS score for distinguishing patients who survived hospital discharge from those who did not, we performed an ROC curve analysis with data from all 44 neonates. From this analysis, the AUC for POS scores was 0.73 (95 % CI, 0.58-0.88), and a cutoff of 55 % for POS had a sensitivity of 74 %, specificity of 64 %, PPV of 61 %, and NPV of 76 % for predicting hospital mortality (Fig. 1a).

**Table 1:** Demographic data, disease characteristics, and therapeutic interventions for neonates with congenital diaphragmatic hernia (CDH)^a^

Characteristic	Hospitalization outcome		*P* value
	Survival (*n* = 25)	Death (*n* = 19)	
Prenatal diagnosis of CDH	9 (36)	9 (47)	0.54
Male sex	16 (64)	11 (58)	0.76
Gestational age, wk	38.6 ± 2.2	37.8 ± 3.4	0.30
Birth weight, kg	3.15 ± 0.67	2.92 ± 0.73	0.29
Small for gestational age	2 (8)	1 (5)	
Apgar score 1 min	6.1 ± 2.6	4.3 ± 2.5	0.02
Apgar score 5 min	6.4 ± 2.5	4.7 ± 1.9	0.02
CDH type			
Left	23 (92)	13 (68)	0.07
Right	2 (8)	5 (26)	
Bilateral	0 (0)	1 (5)	
Liver-up	8 (32)	16 (84)	<0.001
Probability of survival score, %	64 ± 27	45 ± 19	0.01
High (67 %-100 %)	13 (52)	3 (16)	
Moderate (34 %-66 %)	9 (36)	11 (58)	
Low (0 %-33 %)	3 (12)	5 (26)	
Pulmonary hypertension^b^	13 (52)	18 (95)	0.003
Diaphragmatic agenesis	0 (0)	4 (21)	0.03
Peritoneal sac present	4 (16)	0 (0)	0.12
Inhaled nitric oxide	11 (44)	19 (100)	<0.001
Surfactant administration	4 (16)	12 (63)	0.002
Vasoactive support	24 (96)	19 (100)	>0.99
Surgical repair^c^			
Primary closure	23 (92)	3 (43)	0.01
Patch repair	2 (8)	3 (43)	
Muscle flap repair	0 (0)	1 (14)	

^a^Continuous data are presented as mean ± SD; categorical data as number of patients (percentage of sample); ^b^Diagnosed with Doppler echocardiography; ^c^Twelve patients died before surgery; 7 died after surgical repair

### Blood gas analyses

Capillary blood gas data at admission and during the first 24 h following admission were summarized separately for survivors and nonsurvivors and separately for patients who survived at least 24 h and who died within 24 h (Table 2). Predictors of mortality at admission were lower capillary PcO_2_/FIO_2_ ratio, higher PcCO_2_, and lower pH. At 24 h predictors of mortality were lower PcO_2_/FIO_2_ ratio and higher PcCO_2_ (Table 2). Sampling site for all blood gas measurements was preductal (right-hand).

**Table 2:** Preductal capillary blood gas data at admission and during the first 24 h after admission^a^

Characteristic	Hospitalization outcome				*P *value^b^
	Survival (*n* = 25)^d^		Death (*n* = 19)		
	N	Mean ± SD	N	Mean ± SD	
Admission blood gas					
PcO_2_, mm Hg	25	54.9 ± 20.9	19	45.5 ± 18.6	0.13
PcO_2_/FIO_2_, mm Hg	25	72.2 ± 37.3	19	50.3 ± 30.2	0.04
PcCO_2_, mm Hg	25	70.1 ± 25.2	19	91.3 ± 31.1	0.02
pH	24	7.15 ± 0.19	17	7.01 ± 0.19	0.03
Base deficit, mEq/L	24	−5.20 ± 6.92	17	−9.92 ± 8.57	0.058
Postadmission blood gas (≤24 h)^c^					
Highest PcO_2_, mm Hg					
Alive at 24 h	24	87.0 ± 36.7	12	70.6 ± 21.1	0.16
Death at ≤24 h	---	---	7	65.7 ± 19.6	---
Highest PcO_2_/FIO_2_, mm Hg					
Alive at 24 h	24	140.5 ± 65.2	12	75.2 ± 23.8	0.002
Death at ≤24 h	---	---	7	67.8 ± 19.7	---
Highest PcCO_2_, mm Hg					
Alive at 24 h	24	73.3 ± 25.0	12	100.1 ± 34.6	0.01
Death at ≤24 h	---	---	6	117.7 ± 26.7	---
Lowest pH					
Alive at 24 h	23	7.14 ± 0.19	10	7.01 ± 0.20	0.09
Death at ≤24 h	---	---	6	6.88 ± 0.09	---
Highest base deficit, mEq/L					0.131
Alive at 24 h	23	−6.35 ± 6.71	10	−10.42 ± 7.43	---
Death at ≤ 24 h	---	---	5	−17.92 ± 3.66	---

^a^All blood gas data presented in this table were obtained before surgical intervention. ^b^ *P* values are from the 2-sample *t-*test comparing the given blood gas measurement between patients who survived hospitalization and patients who did not. ^c^Comparison of the gas exchange over the first 24 h are restricted to patients who survived at least 24 h. ^d^In the survivor group, one neonate had admission data, but missing information for postadmission blood gases. For this reason the sample sizes for the survival group are one less in the postadmission analysis than the admission analysis. Abbreviations: FIO_2_, fraction of inspired oxygen; PcCO_2_, partial pressure of carbon dioxide in the capillary blood; PcO_2_, partial pressure of oxygen in the capillary blood

### Association of neonatal survival with preductal PcCO_2_

High PcCO_2_ on admission was a strong predictor of mortality. Among survivors, mean PcCO_2_ on admission was significantly lower than that for nonsurvivors (70.1 ± 25.2 vs. 91.3 ± 31.1 mmHg, *P* = 0.02). For survivors, PcCO_2_ remained similar after admission while among nonsurvivors, the PcCO_2_ increased (Table 2). Fig. 1b shows the ROC curve for admission PcCO_2_ to predict mortality calculated with the data for all neonates. From this analysis, the AUC was 0.74 (95 % CI, 0.58-0.89), and a cutoff of 81.8 mmHg for admission PcCO_2_ had a sensitivity of 63 %, specificity of 80 %, PPV of 71 %, and NPV of 74 % for predicting hospital mortality.

### Association of neonatal survival with preductal pH

Low pH on admission was also a predictor of mortality. Among survivors, mean pH on admission was higher than among nonsurvivors (*P* = 0.03; Table 2). From ROC analysis, the AUC was 0.70 (95 % CI, 0.53-0.88), and a cutoff of 7.06 for admission pH had a sensitivity of 65 %, specificity of 75 %, PPV of 65 %, and NPV of 75 % for predicting hospital mortality.

### Association of neonatal survival with admission preductal PcO_2_/FIO_2_

Low *PcO*_*2*_*/FIO*_*2*_ on admission was also a predictor of mortality. Among survivors, mean admission *PcO*_*2*_*/FIO*_*2*_ was 72.2 ± 37.3 mmHg, and among nonsurvivors 50.3 ± 30.2 mmHg (*P* = 0.04; Table 2). From ROC analysis, the AUC was 0.69 (95 % CI, 0.53-0.85), and a cutoff of 59 % for admission *PcO*_*2*_*/FIO*_*2*_ had a sensitivity of 79 %, specificity of 56 %, PPV of 56 %, and NPV of 74 % for predicting hospital mortality.

### Association of neonatal survival from postadmission preductal PcO_2_/FIO_2_

Among patients who survived at least 24 h, the highest recorded PcO_2_/FIO_2_ was higher among survivors than among nonsurvivors (140.5 ± 65.2 mmHg vs. 75.2 ± 23.8 mmHg, respectively, *P* = 0.002). Among hospital survivors, PcO_2_/FIO_2_ increased during the first 24 h of treatment (from 72.2 ± 37.3 mmHg *to* 140.5 ± 65.2 mmHg, for admission vs. highest value in first 24 h, *P* < 0.001). Of those who died after 24 h, PcO_2_/FIO_2_ did not change significantly over the first 24 h (50.3 ± 30.2 vs. 75.2 ± 23.8 mmHg, *P* = 0.104). The predictive value of highest PcO_2_/FIO_2_ ratio over 24 h determined from ROC analysis found the AUC was 0.87 (95 % CI, 0.75-0.98), and a cutoff of 100 mmHg for PcO_2_/FIO_2_ had a sensitivity of 92 %, specificity of 75 %, PPV of 65 %, and NPV of 95 % for predicting hospital mortality (Fig. 1c). For the 7 neonates who did not survive 24 h and were therefore excluded from this analysis, the highest recorded PcO_2_/FIO_2_ ranged from 36 mmHg to 88 mmHg.

### Association of neonatal survival from other vital signs

We also examined the predictive role of other vital signs. The lowest body temperature and lowest mean blood pressure over the first 12 h following admission were significantly lower in patients who died than in those who did not (35.7 °C ± 0.5 °C vs. 36.3 °C ± 0.7 °C, *P* = 0.005; 31.5 ± 10.1 mmHg vs. 39.3 ± 6.1 mmHg, *P* = 0.003).

## Discussion

In our series of 44 CDH high-risk outborn neonates, a significant prognosticating factor for mortality was the inability to improve pulmonary gas exchange. Specifically, outborn neonates without a substantial increase in PcO_2_/FIO_2_ ratio while on mechanical ventilation during the first 24 h following admission had increased mortality. Furthermore, high admission and postadmission PcCO_2_ predicted mortality. This is in agreement with the recent finding by Abbas et al. [19] who reported that inborn CDH nonsurvivors were characterized by inability to maintain sufficient pulmonary gas exchange despite resuscitation.

Up to 25 % mortality among children with CDH is related directly to the degree of lung hypoplasia. The extent of lung hypoplasia can be approximated from the level of oxygenation and ventilation, and both have a linear correlation with hypoplasia [11]. In predicting survival of patients with CDH, several authors have used blood gas analyses [6, 15, 19, 20]. Salas et al. [21] demonstrated that an admission PaCO_2_ greater than 88 mmHg predicts mortality, and an admission PaCO_2_ less than 66 mmHg was associated with improved survival. CDH nonsurvivors were unable to maintain adequate gas exchange over the first 24 h of resuscitation [19]. These findings are consistent with our results. Similarly, Khmour et al. [15] demonstrated that during the era of protective ventilation, both admission PaCO_2_ and preductal oxyhemoglobin saturation obtained within 1 h of admission may predict survival among neonates with CDH. Specifically, admission preductal oxyhemoglobin saturation less than 85 % had a PPV for mortality of 0.82 [15]. Yoder et al. [20] reported a PPV of 57 % to predict mortality when the minimum PaCO_2_ was 70 mmHg or more during the first 24 h and a PPV of 77 % when the best preductal oxyhemoglobin saturation was less than 85 %. A PPV of 88 % for mortality was reported when both parameters were met.

For our neonates who survived the first 24 h, the highest PcO_2_/FIO_2_ ratio was a very good predictor of hospital outcome, with an AUC of 0.87, and a cutoff of 100 mmHg for PcO_2_/FIO_2_ had a PPV of 65 % and an NPV of 95 % for predicting hospital mortality. Our score, obtained from capillary blood gas, is similar to that proposed by the WHSRPF that uses arterial blood gases during the initial 24 h of life. Their prediction score (highest PaO_2_ minus highest PaCO_2_) had AUC of 0.87 with a PPV of 82 % and an NPV of 88 % [7].

All our neonates were transferred from either local or remote hospitals. The most typical ventilatory support during travel was a self-inflating bag and, less frequently, transport ventilators. Because of this, a proportion of neonates in this cohort probably received suboptimal ventilation. This was the basis for our hypothesis that the institution of proper mechanical ventilation upon admission may separate “responders” (those with recruitable lung units) from “non-responders” (those with more severe pulmonary hypoplasia). Based on that premise, one would expect to find the admission blood gas values to be less predictive of outcome than the blood gases following treatment. Indeed, we found that the highest PcO_2_/FIO_2_ achieved over 24 h of treatment represents a better approach in predicting outcome. Furthermore, inefficient ventilation, as inferred from inability to reduce PcCO_2_, was a good prognosticating indicator for mortality.

The limitation of this study is related to its retrospective design, and evaluation of a relatively small number of patients admitted to a single large academic center. Proper interpretation of blood gas analyses may be complex when blood gases are not obtained in a standardized fashion (arterial vs. capillary). Instead of using arterial blood, UHC used capillary blood to obtain measurements of PO_2_, PCO_2_, and pH. A recent meta-analysis showed that blood sampled from the fingertip accurately reflects PaCO_2_ and arterial pH over a wide range of values [18]. Given the existence of high correlation for PCO_2_ and pH between capillary and arterial samples, our findings of association between capillary PcCO_2_ and pH and survival likely indicate that obtaining arterial blood gases would have similar association. In contrast, PcO_2_ less accurately approximates PaO_2_, but the accuracy improves in hypoxic conditions [18], and the admission PcO_2_ in our neonates was lower than normal. While we do not know how accurately PcO_2_ in our study approximated PaO_2_, it is likely that the increase in PcO_2_/FIO_2_ represents an improvement in oxygenation. We have shown that an increase in this ratio per se prognosticates outcome comparably to WHSRPF prediction formula [7]. However, the cutpoints and sensitivity/specificity estimates obtained from our ROC analysis for PO_2_*apply only to blood gases from capillary samples*. Furthermore, because contemporary ventilatory strategies for children with CDH allow for mild hypercapnia, values of PaCO_2_ less than 60–65 mmHg have little meaning for outcome prognostication (e.g., neonates with PaCO_2_ of 45 mmHg or 65 mmHg may reflect the fact that different providers may allow for different levels of hypercapnia); however, high PaCO_2_ values (>80 mmHg) are universally undesirable and are undoubtedly markers of severe lung hypoplasia and increased mortality. Furthermore, the long study period used in our investigation may encompass therapeutic advances which could have changed outcomes in neonates over time. We did not detect a significant temporal change in survival and we do not believe any substantial improvements in practice occurred in our institution during the study period (ECMO was unavailable throughout the entire study period, and protective ventilatory strategies, the use of iNO, and HFOV were available). Finally, our results may lack generalizability to settings with more sophisticated transport management capabilities and institutions with ECMO availability.

## Conclusion

Using a unique population of high-risk CDH outborns with early respiratory distress, we found that the inability to improve gas exchange within 24 h of the initiation of treatment is an ominous sign. Of all the blood gas parameters considered, the magnitude of increase in oxygenation in response to treatments was the best predictor of survival. Therefore, we suggest that improvement of oxygenation during the first 24 h, along with other relevant clinical signs, may help when making decisions regarding survivability or the use of ECMO when available.

## Abbreviations

AUC, area under the curve; CDH, congenital diaphragmatic hernia; CDHSG, Congenital Diaphragmatic Hernia Study Group; ECMO, Extracorporeal membrane oxygenation; FIO_2_, fraction of inspired oxygen; NICU, neonatal intensive care unit; NPV, negative predictive value; PcCO_2_, partial pressure of carbon dioxide in the capillary blood; PcO_2_, partial pressure of oxygen in the capillary preductal blood; POS, probability of survival; PPV, positive predictive value; ROC, receiver operating characteristic; UHC, University Hospital Centre; WHSRPF, Wilford Hall/Santa Rosa clinical prediction formula

## References

[1] Bohn DJ, James I, Filler RM, Ein SH, Wesson DE, Shandling B, Stephens C, Barker GA (1984). The relationship between PaCO2 and ventilation parameters in predicting survival in congenital diaphragmatic hernia. J Pediatr Surg.

[2] O’Rourke PP, Vacanti JP, Crone RK, Fellows K, Lillehei C, Hougen TJ (1988). Use of the postductal PaO2 as a predictor of pulmonary vascular hypoplasia in infants with congenital diaphragmatic hernia. J Pediatr Surg.

[3] Downard CD, Jaksic T, Garza JJ, Dzakovic A, Nemes L, Jennings RW, Wilson JM (2003). Analysis of an improved survival rate for congenital diaphragmatic hernia. J Pediatr Surg.

[4] Bohn D (2002). Congenital diaphragmatic hernia. Am J Respir Crit Care Med.

[5] Kays DW, Langham MR, Ledbetter DJ, Talbert JL (1999). Detrimental effects of standard medical therapy in congenital diaphragmatic hernia. Ann Surg.

[6] Wung JT, Sahni R, Moffitt ST, Lipsitz E, Stolar CJ (1995). Congenital diaphragmatic hernia: survival treated with very delayed surgery, spontaneous respiration, and no chest tube. J Pediatr Surg.

[7] Schultz CM, DiGeronimo RJ, Yoder BA (2007). Congenital diaphragmatic hernia: a simplified postnatal predictor of outcome. J Pediatr Surg.

[8] Congenital Diaphragmatic Hernia Study Group (2001). Estimating disease severity of congenital diaphragmatic hernia in the first 5 minutes of life. J Pediatr Surg..

[9] Richardson DK, Corcoran JD, Escobar GJ, Lee SK (2001). SNAP-II and SNAPPE-II: Simplified newborn illness severity and mortality risk scores. J Pediatr.

[10] Skarsgard ED, MacNab YC, Qiu Z, Little R, Lee SK (2005). SNAP-II predicts mortality among infants with congenital diaphragmatic hernia. J Perinatol.

[11] Germain JF, Farnoux C, Pinquier D, Cortez A, Hartmann JF, Sibony O, de Lagausie P, Beaufils F (1996). Can blood gas values predict pulmonary hypoplasia in antenatally diagnosed congenital diaphragmatic hernia?. J Pediatr Surg.

[12] Park HW, Lee BS, Lim G, Choi YS, Kim EA, Kim KS (2013). A simplified formula using early blood gas analysis can predict survival outcomes and the requirements for extracorporeal membrane oxygenation in congenital diaphragmatic hernia. J Korean Med Sci.

[13] Dibbins AW, Wiener ES (1974). Mortality from neonatal diaphragmatic hernia. J Pediatr Surg.

[14] Mishalany HG, Nakada K, Woolley MM (1979). Congenital diaphragmatic hernias: eleven years’ experience. Arch Surg.

[15] Khmour AY, Konduri GG, Sato TT, Uhing MR, Basir MA (2014). Role of admission gas exchange measurement in predicting congenital diaphragmatic hernia survival in the era of gentle ventilation. J Pediatr Surg.

[16] Bojanic K, Pritisanac E, Luetic T, Vukovic J, Sprung J, Weingarten TN, Carey WA, Schroeder DR, Grizelj R (2015). Survival of outborns with congenital diaphragmatic hernia: the role of protective ventilation, early presentation and transport distance: a retrospective cohort study. BMC Pediatr..

[17] Harrison AM, Lynch JM, Dean JM, Witte MK (1997). Comparison of simultaneously obtained arterial and capillary blood gases in pediatric intensive care unit patients. Crit Care Med.

[18] Zavorsky GS, Cao J, Mayo NE, Gabbay R, Murias JM (2007). Arterial versus capillary blood gases: a meta-analysis. Respir Physiol Neurobiol.

[19] Abbas PI, Cass DL, Olutoye OO, Zamora IJ, Akinkuotu AC, Sheikh F, Welty SE, Lee TC (2015). Persistent hypercarbia after resuscitation is associated with increased mortality in congenital diaphragmatic hernia patients. J Pediatr Surg.

[20] Yoder BA, Lally PA, Lally KP (2012). Does a highest pre-ductal O(2) saturation <85 % predict non-survival for congenital diaphragmatic hernia?. J Perinatol.

[21] Salas AA, Bhat R, Dabrowska K, Leadford A, Anderson S, Harmon CM, Ambalavanan N, El-Ferzli GT (2014). The value of Pa(CO2) in relation to outcome in congenital diaphragmatic hernia. Am J Perinatol.

